# Early Adolescent Friendship Selection Based on Externalizing Behavior: the Moderating Role of Pubertal Development. The SNARE Study

**DOI:** 10.1007/s10802-016-0134-z

**Published:** 2016-02-20

**Authors:** Aart Franken, Mitchell J. Prinstein, Jan Kornelis Dijkstra, Christian E. G. Steglich, Zeena Harakeh, Wilma A. M. Vollebergh

**Affiliations:** 1 Utrecht Centre for Child and Adolescent Studies, Utrecht University, PO Box 80.140, 3508 TC Utrecht, The Netherlands; 2 University of North Carolina at Chapel Hill, Chapel Hill, NC USA; 3 University of Groningen, Groningen, Netherlands

**Keywords:** Alcohol use, Delinquency, Pubertal development, Social network analysis, SIENA, Tobacco use

## Abstract

This study examined friendship (de-)selection processes in early adolescence. Pubertal development was examined as a potential moderator. It was expected that pubertal development would be associated with an increased tendency for adolescents to select their friends based on their similarities in externalizing behavior engagement (i.e., delinquency, alcohol use, and tobacco use). Data were used from the first three waves of the SNARE (Social Network Analysis of Risk behavior in Early adolescence) study (*N* = 1144; 50 % boys; *M*_*age*_ = 12.7; *SD* = 0.47), including students who entered the first year of secondary school. The hypothesis was tested using Stochastic Actor-Based Modeling in SIENA. While taking the network structure into account, and controlling for peer influence effects, the results supported this hypothesis. Early adolescents with higher pubertal development were as likely as their peers to select friends based on similarity in externalizing behavior and especially likely to remain friends with peers who had a similar level of externalizing behavior, and thus break friendship ties with dissimilar friends in this respect. As early adolescents are actively engaged in reorganizing their social context, adolescents with a higher pubertal development are especially likely to lose friendships with peers who do not engage in externalizing behavior, thus losing an important source of adaptive social control (i.e., friends who do not engage in externalizing behavior).

**Electronic supplementary material:**

The online version of this article (doi:10.1007/s10802-016-0134-z) contains supplementary material, which is available to authorized users.

It has been well-established that the dramatic increase in youths’ externalizing behaviors (e.g., delinquency, alcohol use, tobacco use) over the adolescent transition period is strongly associated with social peer processes (e.g., Brechwald and Prinstein 2011; Dishion and Tipsord 2011; Moffitt 2007; Veenstra et al. 2013). Social network theories suggest that early adolescents and their friends’ engagement in similar behaviors are due to two simultaneous peer socialization processes. First, *selection effects* suggest that youth tend to befriend peers who engage in similar levels of behaviors. Second, *influence effects* suggest that adolescents tend to adapt their behavior to become more similar to their friends (for more details see Steglich et al. 2010). Interestingly, although there has been an increasing focus on factors that may mediate or moderate influence effects, still little is known regarding the factors that may make youth more (or less) likely to select friends with similar behavioral proclivities (Veenstra et al. 2013).

Selection effects have substantial potential implications for understanding adolescent social and behavioral development. In particular, selection effects may establish a pattern of person-environment transactions that have implications for both social relationships and for longer-term adjustment. By engaging in specific behaviors (e.g., externalizing behaviors) some adolescents are afforded new social opportunities (i.e., formation of new relationships), or are able to maintain existing relationships with peers (e.g., Moffitt 1993, 2007). Conversely, de-selection effects can lead to dissolution of friendships. In other words, adolescents may break friendship ties with peers who are dissimilar to themselves (e.g., DeLay et al. 2013; Van Zalk et al. 2010). Less is known about the process of de-selection. Findings of studies investigating deselection indicate that deselection of friends who are dissimilar in tobacco use tends to happen among late adolescents who use tobacco (DeLay et al. 2013), and that among 14 year olds selection rather than de-selection is important to explain similarity in delinquency and alcohol use (Van Zalk et al. 2010). Hence, it is important to take both selection and de-selection effects into account. This might be especially important for externalizing behavior, as retention of friendships with peers who do not engage in externalizing behavior may confer a variety of adjustment benefits (Richmond et al. 2012), while more stable friendships with externalizing peers might increase the spread of externalizing behavior (Laursen et al. 2012). Thus, selection is a dynamic process between adolescents’ behaviors and the navigation of their social relationships; by choosing to drink alcohol, smoke cigarettes, or engage in delinquent acts, adolescents are actively engaged in reorganizing their social context (Dishion 2013). An initial step for understanding these processes is to more thoroughly examine factors that impact (de-)selection effects.

Selection effects based on externalizing behaviors may be critical to examine in the early adolescent period for at least two reasons. First, externalizing behavior becomes especially appealing to early adolescents as it might allow them to bridge the *maturity gap* (Moffitt 1993, 2007). Adolescents experience this maturity gap when they feel biologically mature, but society does not grant them adult rights and responsibilities. Adolescents experiencing the maturity gap may be likely to engage in perceived ‘adult-like’ behaviors, such as in externalizing behavior (Moffitt 1993, 2007). Second, brain maturation during early adolescence is associated with increased susceptibility to social rewards before cognitive control is fully developed (e.g., Blakemore and Mills 2014; Crone and Dahl 2012; Prinstein and Giletta 2016; Somerville 2013). The desire to attract such rewarding friends may be especially powerful in early adolescence. For these reasons, early adolescence may be an important period for understanding selection effects based on externalizing behaviors.

Substantial prior research indicates that early adolescents select friends based on similarity in externalizing behaviors, such as delinquent activities, alcohol use, and tobacco use (Burk et al. 2012; Huisman and Bruggeman 2012; Kerr et al. 2012; Light et al. 2013; Mercken et al. 2009; Mercken et al. 2012; Osgood et al. 2013; Steglich et al. 2012). However, not all adolescents are equally likely to do so. Moreover, findings regarding friendship selection on externalizing behavior have been inconsistent (e.g., Weerman 2011). Weerman (2011) provides several explanations for the lack of selection effects in some past work. For instance, studies using two measurement waves may not be sufficient to detect effects. Moreover, Weerman (2011) notes that selection effects might take place in smaller rather than in larger multiple grade-level friendship networks. Alternatively, there might have been other factors that moderate friendship selection effects (see also Veenstra et al. 2013). The current study uses three waves of grade wide nomination data to assess pubertal development as a potential moderator.

Pubertal development might be relevant to selection and de-selection effects, yet it has been understudied and has not been studied as a moderating variable using models simultaneously estimating (de-)selection and influence effects. Pubertal development precipitates the experience of the maturity gap (Moffitt 1993), as well as an increased susceptibility to social rewards, such as those that come from friendship (e.g., Blakemore and Mills 2014; Crone and Dahl 2012; Somerville 2013). Moreover, early pubertal development is generally considered a risk factor for the development of externalizing behavior among both boys and girls (for a review, see Negriff and Susman 2011). Preliminary results suggest that among boys with more advanced levels of pubertal development, friends’ externalizing behavior is associated with boys’ own externalizing behavior, while this is not the case for boys with a less advanced pubertal development (Felson and Haynie 2002). Westling and colleagues (Westling et al. 2008) found that the association between pubertal development and externalizing behavior (including alcohol and tobacco use) was moderated by affiliation with deviant peers for girls and not for boys. Furthermore, among early developing girls, older friends might be important for the development of externalizing behaviors such as delinquency (see Stattin et al. 2011). Last, friends’ delinquency affects the association between early pubertal development and externalizing behaviors (i.e., delinquency) for boys and girls in a similar way and it is therefore possible to study these effects for boys and girls simultaneously (Lynne et al. 2007). Thus, it can be expected that early adolescents with more advanced pubertal development are especially interested in the social rewards associated with externalizing behavior for both boys and girls and may therefore be more likely to select friends based on similarity in externalizing behavior tendencies.

This study examined pubertal development as a moderator of (de-)selection effects while addressing several limitations of prior work (see Veenstra et al. 2013). To stringently examine the associations between early adolescents’ externalizing behavior and friendship selection, it is important to ensure that 1) associations are not inflated due to shared method variance; 2) selection and effects are parsed from overall network effects (i.e., controlling for cohort-wide changes in friendship selection); 3) both selection and de-selection effects are modeled separately. Each of these issues is addressed using Stochastic Actor-Based Modeling (SABM). This statistical technique takes friendship structures into account while investigating the co-evolution of friendship and behaviors. Moreover, SABM disentangles longitudinal friendship selection, de-selection, and influence processes.

It was hypothesized that higher levels of adolescents’ pubertal development would be associated with a stronger tendency to select friends based on similar levels of externalizing behavior, and to de-select friends based on different levels of externalizing behavior. Data from the SNARE (Social Network Analysis of Risk behavior in Early adolescence) study were used to examine these hypotheses. A unique strength of this dataset is the opportunity to examine friendship networks of two cohorts in the first year of secondary school, starting at age 12, in each of two schools in the Netherlands, thus enabling the examination of selection of friends in an largely unacquainted network. The study of the same hypothesis across all four social networks allows for an examination of internal replication of findings.

## Methods

### Procedure and Participants

Participants included 1144 students from the first year of secondary school (i.e., similar to 7th grade in the US; 50 % boys), aged 11.1 till 15.6 (*M* = 12.7, SD = 0.47). A total of 97 % of participants were born in the Netherlands (as were 87 % of their fathers and 88 % of their mothers).

The SNARE study is an ongoing prospective cohort study involving schools in two regions of the Netherlands; ethical approval for the study was granted by the first author’s university. Participants were recruited in their first or second grade of school (i.e., similar to 7th-8th grades in the US) in Year 1. In Year 2, a second cohort was added, including students in first grade at the same schools. A passive consent procedure was used; students or their parents were asked to send a reply card or email within 2 weeks, it they wished to refrain from participation. In total 1826 students participated in the SNARE study, and 40 students (2.2 %) refused to participate.

Data from the first three waves of data collection were available for analysis. As the focus was on the development of externalizing behavior during early adolescence, only data from the 1444 first grade students were used. Of the participants, 46.1 % followed lower level education (including preparatory secondary school for technical and vocational training) and 53.9 % followed higher level education (including preparatory secondary school for higher professional education and university). As intergenerational mobility in education is rather low in the Netherlands (see Van de Werfhorst et al. 2001), this indicates that a bit less than half of the participants came from relatively lower socioeconomic status background, and a bit more than half of the participants came from higher socioeconomic status background. The first assessment took place in October (Time 1), the second in December (Time 2), and the third in April (Time 3). During each assessment, participants completed study questionnaires on the computer while a teacher and research assistant were present. The percentage of missing participants, who did not agree to participate or were absent during data collection, in the four friendship networks ranged between 1 and 5 % (see Table 2). At Time 1 participants who were absent or indicated not to take part in the study were (if data was available) compared to their peers who were not absent. Absent participants did not differ (*p* > 0.05) from their peers in gender (53 % were boys, versus 50 % of the present participants), their received friendship nominations (25.0 % of their classmates selected them as friends, compared to 24.8 % of the present participants), educational level (50 % followed higher level education, compared to 53.9 % of the present participants). However, they were slightly older (*p* < 0.05; absent participants had an average age of 12.8 years, while the present participants had an average age of 12.7 years). Peer nominations were completed using CS socio software (www.sociometric-study.com). Friendship nominations were conducted by asking participants to select an unlimited number of their closest same- or cross-gender friends from a roster of all classmates, presented in alphabetical order, starting with a random name. Participants were permitted to list peers outside their classroom, using a search function.

### Measures

#### Self-reported Externalizing Behaviors (Time 1 – Time 3)

At all three time points, participants reported their engagement in three forms of externalizing behavior, including delinquent behavior, alcohol use, and tobacco use. *Delinquent behavior* was measured with 17 items by asking participants how often (between *0* and *12 or more times*) they had been involved in 17 types of delinquent behavior during the last month; including stealing, vandalism, burglary, violence, weapon carrying, threatening to use a weapon, truancy, contact with the police, and fare evasion in public transport (e.g., Nijhof et al. 2010; Van der Laan et al. 2010). For *alcohol use*, participants used a 13 point scale (ranging from *0* to *over 40 times*) to report on how many occasions they consumed alcohol in the last month (Wallace et al. 2002). For *tobacco use*, participants used a 7-point scale (ranging from *never* to *more than 20*) to indicate how many cigarettes they smoked daily over the past month (e.g., Monshouwer et al. 2011). Because data using continuous measures of externalizing behavior frequency were highly skewed (see Table 1), all externalizing behavior data were recoded as binary, indicating no engagement at all (0) or any engagement (1) in delinquent behavior, alcohol use, and tobacco use. This recoding allowed for an examination of selection effects based on externalizing behavior engagement rather than the frequency of externalizing behavior engagement. An exploratory factor analysis (using maximum likelihood estimations and oblique rotation) tested if the binary-coded externalizing behaviors loaded on a single factor; they loaded on one factor, explaining 55.3 % of the variance (similar results were obtained with the continuous scores, explaining 61.4 % of the variance). Therefore, a composite variable, representing the number of different externalizing behaviors participants engaged in (i.e., delinquency, alcohol, or tobacco use), was computed; resulting in scores between zero (no externalizing behaviors) and three (all externalizing behaviors).

**Table 1:** Overview of the number of participants scoring 0, 1, or higher than 1 on delinquency, alcohol use, and tobacco use; at Time 1, Time 2, and Time 3

	School 1		School 2	
	Cohort 1	Cohort 2	Cohort 1	Cohort 2
	Participants	Participants	Participants	Participants
Delinquency Time 1				
0	332 (78.3 %)	273 (72.6 %)	135 (78.6 %)	95 (70.9 %)
1	47 (11.1 %)	44 (11.7 %)	14 (8.1 %)	25 (18.7 %)
>1	45 (10.6 %)	59 (15.7 %)	23 (13.3 %)	14 (10.4 %)
Delinquency time 2				
0	316 (75.1 %)	268 (72.6 %)	139 (79.4 %)	95 (71.4 %)
1	47 (11.2 %)	49 (13.3 %)	12 (6.9 %)	22 (16.5 %)
>1	58 (13.8 %)	52 (14.1 %)	24 (13.7 %)	16 (12.0 %)
Delinquency Time 3				
0	304 (75.1 %)	273 (72.6 %)	128 (73.1 %)	94 (70.7 %)
1	46 (11.4 %)	49 (13.0 %)	24 (13.7 %)	13 (9.8 %)
>1	55 (13.6 %)	54 (14.4 %)	23 (13.1 %)	26 (19.5 %)
Alcohol Time 1				
0	376 (89.3 %)	321 (86.3 %)	161 (93.1 %)	126 (94.7 %)
1	27 (6.4 %)	28 (7.5 %)	6 (3.5 %)	6 (4.5 %)
>1	18 (4.3 %)	23 (6.2 %)	6 (3.5 %)	1 (0.8 %)
Alcohol Time 2				
0	376 (89.3 %)	328 (90.4 %)	161 (92.5 %)	119 (90.8 %)
1	24 (5.7 %)	16 (4.4 %)	8 (4.6 %)	10 (7.6 %)
>1	21 (5.0 %)	19 (5.2 %)	5 (2.9 %)	2 (1.5 %)
Alcohol Time 3				
0	355 (87.9 %)	319 (89.4 %)	154 (89.0 %)	107 (86.3 %)
1	22 (5.4 %)	27 (7.5 %)	8 (4.6 %)	8 (6.5 %)
>1	27 (6.7 %)	15 (4.2 %)	11 (6.4 %)	9 (7.3 %)
Tobacco Time 1				
0	407 (96.2 %)	349 (93.6 %)	171 (98.8 %)	134 (100 %)
1	7 (1.7 %)	9 (2.4 %)	1 (0.6 %)	
>1	9 (2.1 %)	15 (4.0 %)	1 (0.6 %)	
Tobacco Time 2				
0	405 (96.2 %)	343 (93.5 %)	168 (96.0 %)	125 (94.7 %)
1	10 (2.4 %)	9 (2.5 %)	3 (1.7 %)	3 (2.3 %)
>1	6 (1.4 %)	15 (4.1 %)	4 (2.3 %)	4 (3.0 %)
Tobacco Time 3				
0	366 (90.6 %)	332 (89.0 %)	165 (94.8 %)	115 (90.6 %)
1	22 (5.4 %)	11 (2.9 %)	5 (2.9 %)	4 (3.1 %)
>1	16 (4.0 %)	30 (8.0 %)	4 (2.3 %)	8 (6.3 %)

#### Pubertal Development (Time 1, Time 2)

The Pubertal Development Scale (PDS; Petersen et al. 1988) was used to assess pubertal development. The PDS used a 4-point scale, ranging from *not yet started* (0), *recently started* (1), *started a while ago* (2), to *already completed* (3), to measure various indicators of pubertal development. Girls were asked four questions regarding their body grow spurt, body hair (pubic hair), changes in skin (pimples), and breast growth. Girls were allowed to skip the question regarding menarche, as a result there were more missing scores and this question was not included in the current analyses. Boys were asked about their body growth, body hair (pubic hair), skin changes, voice changes, and beard growth. Mean scores were computed for girls and boys separately, resulting in a scale with an acceptable internal consistency at Time 1 (alpha = 0.70 for girls, and 0.79 for boys).

#### Friendship Nominations (Time 1 – Time 3)

Participants were asked to name their best friends. Participants could nominate unlimited friends within their class and, afterwards, friends from their grade. Grade networks were used for the current analyses resulting in four friendship networks (i.e., two schools, two cohorts).

### Analysis Strategy

Preliminary analyses included descriptive statistics for each of the four social networks (i.e., two cohorts in two schools). For each network and each assessment, the average age, percentage of boys, average externalizing behavior level, the number of externalizing behaviors participants engaged in, pubertal development scores, the fraction of missing participants per assessment, and the average number of friends for participants were computed. A Jaccard index, indicating relative network stability over time, also was calculated.

All network analyses were conducted using SIENA (Simulation Investigation for Empirical Network Analyses), version 4, in R. SIENA is actor based, and models the longitudinal co-evolution of social networks and individual characteristics (Ripley et al. 2014). For the social networks, at each time point SIENA reads the presence (identified by the score 1) or absence (identified by the score 0) of friendship ties between participants (actors) in the network, and the number of externalizing behaviors participants engage in. As there were three time points, each network has three friendship network input files and each participant has three scores on externalizing behavior. SIENA also reads two types of individual characteristics, constant or varying characteristics. Constant characteristics do not change over time (such as participants’ gender). Varying characteristics can change over time (such as the pubertal development scale). SIENA uses information about a varying characteristic at Time 1 to estimate changes between Time 1 and Time 2, and information at Time 2 to estimate changes between Time 2 and Time 3.

While controlling for *structural network effects* (i.e., the structure of friendships in the network), SIENA estimates both selection effects *(network dynamics)* and influence effects *(behavior dynamics)* longitudinally. The changes in individual behavior were modeled as an increase or decrease in the number of externalizing behaviors participants engaged in (ranging from zero to three externalizing behaviors). SIENA estimates changes between two points in time. For the current analyses the dependent variables are the network ties (friendships) and the number of externalizing behaviors participants engaged in (delinquent behavior, alcohol use, and tobacco use). The outcomes of SIENA analyses are based on an iterative Markov chain Monte Carlo approach (Snijders et al. 2010; Ripley et al. 2014). SIENA shows t-ratios as convergence statistics for the different effects in the model, with t-ratios below 0.10 signifying good convergence; models with good convergence were used for interpretation of the results. The pubertal development score at Time 1 was used for the analyses in the first period (between Time 1 and Time 2), and the score at Time 2 was used for the analyses in the second period (between Time 2 and Time 3). The effects which were modeled will be described below, for more detail and the equations behind these effects we refer to the SIENA manual (Ripley et al. 2014).

*Structural network effects* commonly used in comparable studies were added (see Ripley et al. 2014; Veenstra et al. 2013) afterwards, using goodness of fit indices, other network effects were added to optimally capture the friendship structure in the current networks. The effects which are generally included in SIENA analyses were network density (^1A^ 1 the number of present versus absent friendship ties in the network), reciprocity (^1B^ the likelihood of reciprocated friendship ties), transitive triplets (^1C^ likelihood to befriend friends of friends), three-cycles (^1D^ indicates generalized reciprocity and negative hierarchies), indegree popularity (^1E^ square root version; likelihood for participants who receive many friendship nominations to receive extra friendship nominations), indegree activity (^1F^ square root version; likelihood for participants who receive many friendship nominations to send extra friendship nominations), and outdegree activity (^1G^ square root version; likelihood for participants who send out many friendship nominations to send out extra friendship nominations); for more details see Ripley et al. (2014). To improve model fit, density and indegree popularity were allowed to vary between assessment periods. Furthermore, transitive reciprocated triplets were modeled to estimate the likelihood for triads (a group of three friends) to reciprocate friendships.

Before examining study hypotheses, several factors potentially affecting the social network (i.e., *network dynamic effects*) were estimated as covariates (see Veenstra et al. 2013). The effects of same-gender friendship selection (^2C^ i.e., girls nominate girls; boys nominate boys; girls were coded as 0, boys as 1) were estimated as well as the effects of proximity by using adolescents’ classroom and school locations as covariates (^2C^ School 1 consisted of four locations). The effects of gender on provision (^2B^ ego) and receipt of (^2A^ alter) friendship nominations also was controlled.

To examine our main hypotheses, we included the effects of pubertal development and externalizing behavior on friendship nominations given (^2B^ ego effects) and received (^2A^ alter effects). Furthermore, we included the selection similarity effect (^2C^) modelling the likelihood of providing and selecting similar friends based on externalizing behavior and pubertal development. Of particular note, two interaction effects were added to examine if pubertal development had an impact on the likelihood for participants to select and deselect friends who were dissimilar in externalizing behavior (maintain (^2D^) and create (^2E^) pubertal development x externalizing behavior similarity selection).

Although not a focus of the current study, SIENA simultaneously examines influence, as well as selection effects. Thus, several *behavior dynamic effects* also were estimated (see Veenstra et al. 2013). Behavior dynamic effects model changes in externalizing behavior. They model the rate of change (externalizing behavior change period 1 & 2), and whether behavior change conforms to linear (externalizing behavior linear shape) or quadratic (externalizing behavior quadratic shape) trends. A main effect of influence is estimated as the likelihood that participants adapt their externalizing behavior to become more similar to the average externalizing behavior of their friends (externalizing behavior influence). The effects of pubertal development on behavior change was also modeled (effects from pubertal development). An interaction effect between pubertal development and externalizing behavior also was examined (pubertal development x externalizing behavior influence) to determine whether susceptibility to peer influence depends on pubertal development. Overall, the inclusion of these effects was similar to prior research using SIENA (e.g., DeLay et al. 2013; Van Zalk et al. 2010).

A meta-analysis of the parameters was conducted on the four networks. This meta-analysis was conducted using the SIENA likelihood based method for meta analyses (for more information see Ripley et al. 2014). Although this analysis is usually not viable for less than 10 networks (Ripley et al. 2014) it worked well with the current networks due to normality of distributions of estimates.

## Results

### Descriptive Statistics of the Networks, and Externalizing Behaviors Within Networks

Table 2 lists descriptive statistics for each of the four networks examined in this study. Results at Time 1 suggested that all four networks did not differ in age or delinquency level. There were some small differences in gender distribution, alcohol use, tobacco use, overall externalizing behavior, and or pubertal development. None of the students of the smallest network, cohort 2 of School 2 used tobacco at Time 1.

**Table 2:** Descriptive statistics of friendship networks for school 1 (Cohort 1 *N* = 432, Cohort 2 *N* = 390) and school 2 (Cohort 1 *N* = 186, Cohort 2 *N* = 136), Times 1, 2 and 3

Variable	School 1				School 2			
	Cohort 1		Cohort 2		Cohort 1		Cohort 2	
	*M*	*(SD)*	*M*	*(SD)*	*M*	*(SD)*	*M*	*(SD)*
Age								
Time 1	12.65	(0.43)	12.65	(0.43)	12.66	(0.48)	12.70	(0.68)
% boys								
Time 1*	0.50^ab^	(0.50)	0.48^a^	(0.50)	0.47^ab^	(0.50)	0.61^b^	(0.49)
Delinquency								
Time 1	0.22	(0.41)	0.27	(0.45)	0.21	(0.41)	0.29	(0.46)
Time 2	0.25	(0.43)	0.27	(0.45)	0.21	(0.41)	0.29	(0.45)
Time 3	0.25	(0.43)	0.27	(0.45)	0.27	(0.44)	0.29	(0.46)
Alcohol								
Time 1*	0.11^ab^	(0.31)	0.14^a^	(0.34)	0.07^b^	(0.25)	0.05^b^	(0.22)
Time 2	0.11	(0.31)	0.10	(0.30)	0.07	(0.26)	0.09	(0.29)
Time 3	0.12	(0.33)	0.14	(0.35)	0.11	(0.31)	0.14	(0.35)
Smoking								
Time 1*	0.06^ab^	(0.31)	0.10^a^	(0.42)	0.01^b^	(0.11)	0.00^b^	(0.00)
Time 2	0.05	(0.28)	0.10	(0.42)	0.04	(0.20)	0.05	(0.22)
Time 3*	0.13^ab^	(0.44)	0.19^a^	(0.56)	0.05^ab^	(0.22)	0.09^b^	(0.29)
Externalizing behavior								
Time 1*	0.38^ab^	(0.77)	0.51^a^	(0.94)	0.29^b^	(0.60)	0.34^ab^	(0.56)
Time 2	0.41	(0.73)	0.45	(0.86)	0.31	(0.66)	0.41	(0.69)
Time 3	0.47	(0.87)	0.59	(1.00)	0.42	(0.71)	0.47	(0.76)
Pubertal development								
Time 1*	0.84^a^	(0.53)	0.93^ab^	(0.58)	0.99^b^	(0.56)	0.88^ab^	(0.55)
Time 2*	0.85^a^	(0.53)	0.90^ab^	(0.59)	0.96^ab^	(0.63)	1.00^b^	(0.56)
Missing fraction								
Time 1	0.01		0.03		0.05		0.01	
Time 2	0.01		0.04		0.03		0.02	
Time 3	0.03		0.03		0.02		0.02	
Average number of friendships connections								
Time 1	7.05		7.65		7.64		6.44	
Time 2	7.93		9.00		7.99		7.66	
Time 3	7.41		8.09		8.05		6.08	
Jaccard index								
Time1 – Time 2	0.46		0.47		0.44		0.45	
Time 2 – Time 3	0.46		0.48		0.44		0.45	

* One-way ANOVA between group differences at *p* < 0.05. Within each time point (i.e., row), *Mean* scores with different superscripts differ significantly from each other at *p* < 0.05; calculated with a post-hoc Tukey Honestly Significant Difference test

Table 2 also includes network characteristics for each cohort. There were between 1 and 5 % absent participants during the assessments. On average, participants had between six and nine friends in all networks and waves. The Jaccard index indicates the relative stability of each network over time. An index between 0.44 and 0.48, indicating the percentage of stable friendships, is well within the desired range for longitudinal social network analyses (Veenstra et al. 2013).

### Effects of Pubertal Development on Friendship Selection of Externalizing Behavior

The outcomes of the SIENA analyses are shown in Table 3. The structural network effects model the network structure, and optimize the goodness of fit of the networks. Network dynamic effects indicate the effects of externalizing behavior, pubertal development, and control variables on selection effects. Main effects were generally consistent with prior research. Participants’ selection of friends was significantly associated with similarity in gender and class. Location was marginally significant in the meta-analysis at *p* = 0.05, probably because the effect was only based on the two networks of school 1. Both of these effects were significant when examined in their respective network (see supplementary materials). Higher levels of participants’ engagement in externalizing behaviors were associated with the provision of more friendship nominations (see positive externalizing behavior sent effect). Participants were more likely to select friends who were similar in their engagement of externalizing behavior (see externalizing behavior similarity), although this effect was marginally significant (*p* = 0.05). Participants also were likely to select friends at similar levels of pubertal development (see pubertal development similarity effect). Behavior dynamics results also revealed a significant negative linear effect, and positive quadratic effect, for changes in externalizing behavior over time.

**Table 3:** Meta-analysis estimates the evaluation functions and standard errors of selection and influence effects for externalizing behavior and pubertal development in friendship, Time 1, 2, and 3

Variable		Meta-analysis
*Network dynamics*		
^1^Outdegree (density) ^1A^	Period 1	−2.31* (0.14)
	Period 2	0.12 (0.12)
Reciprocity ^1B^		2.57* (0.12)
Transitive triplets ^1C^		0.52* (0.02)
Transitive reciprocated triplets ^1D^		−0.43*(0.03)
3-cycles ^1E^		−0.06* (0.02)
Indegree - popularity (sqrt) ^1F^	Period 1	0.05 (0.06)
	Period 2	−0.14* (0.04)
Indegree – activity (sqrt) ^1G^		−0.98* (0.11)
Outdegree – activity (sqrt) ^1H^		0.15* (0.04)
^2^Sex received ^2A^		−0.08 (0.06)
Sex sent ^2B^		−0.14 (0.12)
Sex similarity ^2C^		0.71* (0.05)
Location similarity ^2C^		0.38 (0.03)
Class similarity ^2C^		0.77* (0.07)
Externalizing behavior received ^2B^		0.09 (0.05)
Externalizing behavior sent ^2A^		0.23* (0.05)
Externalizing behavior similarity ^2C^		0.59 (0.19)
Pubertal development received ^2B^		−0.01 (0.02)
Pubertal development sent ^2A^		−0.03 (0.02)
Pubertal development similarity ^2C^		0.37* (0.11)
Pubertal development sent x externalizing behavior similarity maintain ^2D^		0.80* (0.22)
Pubertal development sent x externalizing behavior similarity create ^2E^		−0.45 (0.20)
*Behavior dynamics*		
^3^Externalizing behavior change period 1 ^3A^		1.36* (0.12)
Externalizing behavior change period 2 ^3A^		1.41* (0.15)
Externalizing behavior change linear shape ^3A^		−1.26* (0.07)
Externalizing behavior change quadratic shape ^3A^		0.24* (0.06)
Externalizing behavior influence ^3B^		1.07* (0.21)
Effect from pubertal development ^3C^		0.11 (0.06)
Pubertal development x externalizing behavior influence ^3D^		0.23 (0.29)

*p* < 0.10 * *p* < 0.05. ^1^ effects estimating the structure of the friendship network, for descriptions of single effects see the main text. ^2^ effects estimating friendship selection. ^2A^ received effects estimate the number of received friendship ties for participants with this characteristic. ^2B^ sent effects estimate the number of sent out friendship ties for participants with this characteristic. ^2C^ similarity effects estimate if participants base friendship selection on similarity of this characteristic. ^2D^ interaction assessing the impact of pubertal development on likelihood of maintaining friendships based on externalizing behavior. ^2E^ interaction assessing the impact of pubertal development on likelihood of creating friendships based on externalizing behavior ^3^ effects estimating the change of behavior. ^3A^ estimating the development of externalizing behavior, and if this has a linear or quadratic shape. ^3B^ estimating the effect of this characteristic on the development of externalizing behavior. ^3C^ estimating the effect of the average externalizing behavior of friends on the development of participants’ externalizing behavior. ^3D^ interaction assessing the impact of pubertal development on friends’ influence on the development of participants’ externalizing behavior

**Fig. 1:**
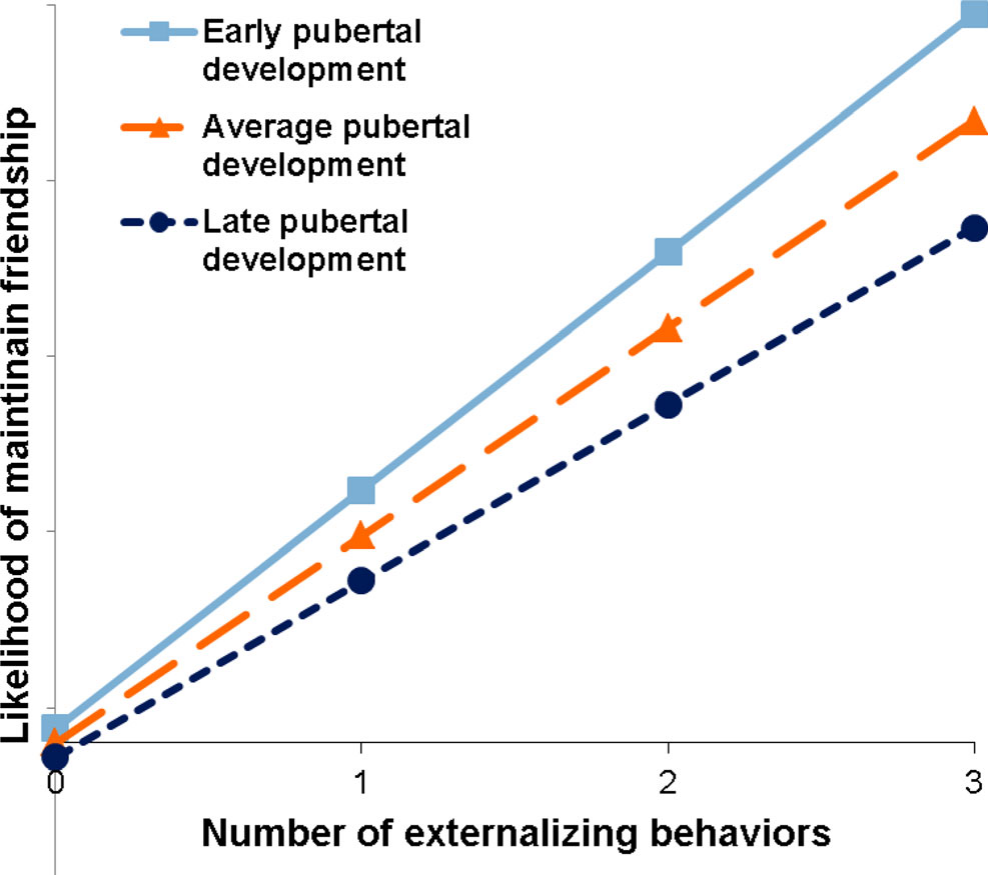
The interaction between pubertal development and maintaining friendships based on similarity in externalizing behavior based on the effects of the meta analysis. The effect is shown in isolation (rather than in context of other effects) for illustrative purposes, the number of externalizing behaviors is shown and the levels of pubertal development are early (+1 *SD*), average (Mean score), and late (-1 *SD*)

Consistent with hypotheses interaction effects (between pubertal development and maintaining friends based on externalizing behavior) emerged, suggesting that over time pubertal development influenced the likelihood to maintain friends who are similar in externalizing behavior. As can be seen in Fig. 1, this effect indicates that among more pubertally developed participants, maintenance of friendship ties over time was more strongly associated with similarity in externalizing behavior especially at higher engagement in externalizing behavior. In other words, adolescents with a higher pubertal development who engage in externalizing behavior were more likely to de-select friends with dissimilar levels of externalizing behavior; there was no difference for adolescents who do not engage in externalizing behavior. However, pubertal development did not influence the likelihood to create friendships based on similarity in externalizing behavior.

Although not a focus of the current study, results also indicated a significant effect for peer influence in externalizing behavior, suggesting that participants’ externalizing behavior became more similar to the average level of their friends’ externalizing behavior over time. Specifically, results indicated that friends’ engagement in more types of externalizing behavior (i.e., delinquency, alcohol, tobacco use) was associated with participants’ adoption of more types of externalizing behavior over time. The direct effect of pubertal development on externalizing behavior nor the interaction between pubertal development and externalizing behavior influence were significant. In other words, participants’ level of pubertal development was not associated with an increase in externalizing behavior over time, nor with differences in adolescents’ propensity for peer influence.

## Discussion

This study focused on friendship (de-)selection processes in early adolescence. Pubertal development was examined as a potential moderator of the relationship between externalizing behavior and friendship (de-)selection. It was hypothesized that more advanced pubertal development would be associated with an increased tendency for adolescents to select their friends based on their similarities in externalizing behavior engagement. This study is unique in its focus on pubertal development as a moderator of selection effects using contemporary approaches for examining peer selection. Results supported the hypotheses. Overall, results indicated that adolescents are especially likely to select peers as friends if those peers are similar in their externalizing behavior engagement; this effect was increasingly evident for deselection of friends by early adolescents with higher levels of adolescents’ pubertal development. Those adolescents with higher pubertal development were more likely to remain friends with peers who have a similar engagement in externalizing behavior, and to break friendship with those who do not.

Results have several important implications for adolescent development. First, findings suggest that more pubertally-developed adolescents are not more inclined to externalizing behavior, but are more likely to lose their friends who do not engage in externalizing behavior, thus they might lose an important source of adaptive social support (e.g., Richmond et al. 2012). Research suggests that adaptive social support, from non-deviant peers, may be especially important for helping adolescents cope with stressors, and for socializing adolescents towards adaptive developmental outcomes such as high academic achievements (Dishion and Tipsord 2011). Findings may elucidate a mechanism by which early-starter adolescent externalizing behavior leads to maladaptation in other domains.

Inversely, results suggest that adolescents who are more pubertally-developed are more likely to have stable friendships with other externalizing youth. In other words, these adolescents may be at greater risk for further deviant peer socialization. Note that findings in this study did not demonstrate that pubertal development was associated with greater susceptibility to peer influence, however. An interesting direction for future work will be the examination of these processes over a wider range of development. It is possible that early adolescents selection processes lead to heightened influence processes later in adolescence that were beyond the assessment window in this study. Alternatively, it is possible that the effects of pubertal development on peer influence processes may be limited to selection effects, and not as relevant for influence effects. Both are intriguing possibilities for future work.

This study revealed several additional interesting results. First, adolescents were likely to select their friends based on similarity in pubertal development, even when taking selection on externalizing behavior into account. Second, although several studies have associated early pubertal development with externalizing behavior during early adolescence (for an overview see Graber et al. 2010), this association might disappear when taking friendship selection and influence processes into account.

This study has several strengths. First, by investigating participants at the start of secondary schools it was possible to capture the beginning of new friendship networks, thus allowing us to study selection effects isolated from previous networks. Second, it was possible to look at generalizability of our findings as we studied four independent but similar whole grade friendship networks; which can be found in the supplementary materials. Furthermore, our study builds on previous studies investigating friendship selection and deselection processes in externalizing behavior among adolescents (e.g., DeLay et al. 2013; Van Zalk et al. 2010) by showing that deselection based on externalizing behavior is important especially among adolescents with a higher pubertal development and engagement in externalizing behavior.

Future studies should address several limitations of this study. First, to examine cohesive social networks, peer nominations emphasized friendships within children’s own school grade. However, deviant peer affiliations in particular may include peers from other grades. Future research allowing cross-grade nominations, and even friendship nominations outside of the school context may reveal additional relevant social influences (Kerr et al. 2007). Although we were able to use a meta-analysis there were some differences between the networks (see the supplementary materials) which merit further investigation. Also Mercken et al. (2009) showed outcomes to differ across several meta-analyses in different countries, our findings add to this study by showing that differences might also occur while comparing networks within the same country or even within the same school. Possibly, power issues due to the dichotomization of our variables (see Markon et al. 2011) might have affected our results; as most significant findings occurred in the larger cohorts examined in this study. Alternatively, differences between the networks might occur because of factors not captured in the current analyses. A recent review (Marschall-Lévesque et al. 2014) pointed out the importance of studying school, neighborhood, and parenting effects when studying the associating between peers and substance use. Parenting effects might be especially important while studying pubertal development (Westling et al. 2008). Therefore, future studies should investigate such factors as potentially affecting the interplay between externalizing behavior, peers, and pubertal development. Third, this study examined an externalizing behavior composite, indicating whether adolescents had engaged in any delinquent behavior, alcohol, or tobacco use. This composite score was used as the data was highly skewed, however the consequence of combining these variables should be further investigated. Moreover, the dichotomized variables were then further collapsed into an ordinal scale. This is an unfortunate, but necessary limitation of SIENA. Identifying alternate approaches for handling continuous measures in SABM is a critical direction for future work. Furthermore, whether these same processes are relevant for the frequency with which adolescents engage in each of these behaviors remains unexplored as we did not conduct analyses at the individual item level. Especially the role of adolescents who engage very frequently in externalizing behaviors might be important to investigate. Future studies could investigate the role of pubertal development in influence and selection processes related to changes in different forms of externalizing behaviors separately, such as delinquency, alcohol use, and tobacco use. Such studies might benefit from investigating slightly older adolescents, when there is a higher occurrence of and change in externalizing behavior. Last, the sophistication of the models examined in this study prohibited a test of more complex gender or family interactions as even larger sample sizes would be needed. Nevertheless, the study of further moderation is a critical future direction.

In conclusion, this study suggested that during early adolescence pubertal development plays a pivotal role in adolescents’ friendship selection. The implications of externalizing behavior engagement on adolescents’ social network has been relatively under-explored, but could be a useful direction to consider whether providing psychological services to at-risk youth. Especially pubertally more advanced adolescents should be supported in maintaining friendships with peers who do not engage in externalizing behavior, as such friends provide an important social support network (Richmond et al. 2012).

## Electronic supplementary material

Below is the link to the electronic supplementary material.

ESM 1 (DOC 46 kb)

## Footnotes

1 Superscripts in the text correspond with the effects which can be found in Table 3.
