# Supplementary material for: Early Adolescent Friendship Selection Based on Externalizing Behavior: the Moderating Role of Pubertal Development. The SNARE Study
Source: J Abnorm Child Psychol. 2016 Feb 20;44(8):1647–57. doi: 10.1007/s10802-016-0134-z (PMC5061845; doi:10.1007/s10802-016-0134-z)
Supplement: Supplementary file 1 — (DOC 46 kb) [file 10802_2016_134_MOESM1_ESM.doc]

Table 4

*Estimates Evaluation Functions and Standard Errors of Selection and Influence Effects for Externalizing Behavior and Pubertal Development in Friendship Networks for Two Schools, Two Cohorts, Time 1, 2, and 3*

Variable School 1 School

Cohort 1 Cohort 2 Cohort 1 Cohort 2

*N 432 390 186 136*

*Network Dynamics*

^1^ Outdegree (density) ^1A^ Period 1 -2.12* (0.24) -2.44* (0.23) -2.53* (0.29) 0.54 (1.26)

Period 2 0.08 (0.21) 0.27 (0.21) 0.11 (0.25) -0.49 (0.51)

Reciprocity ^1B^ 2.90* (0.11) 2.39* (0.09) 2.36* (0.17) 2.63* (0.19)

Transitive triplets ^1C^ 0.56* (0.02) 0.49* (0.02) 0.47* (0.03) 0.57* (0.05)

Transitive reciprocated triplets ^1D^ -0.52* (0.03) -0.38* (0.03) -0.43* (0.05) -0.36* (0.07)

3-cycles ^1E^ -0.04 (0.02) -0.10* (0.02) -0.03 (0.04) -0.07 (0.06)

Indegree - popularity (sqrt) ^1F^ Period 1 0.08* (0.03) -0.08* (0.03) 0.21* (0.04) -0.01 (0.08)

Period 2 -0.11 (0.07) -0.22* (0.07) -0.07 (0.08) -0.20 (0.15)

Indegree – activity (sqrt) ^1G^ -1.10* (0.14) -0.76* (0.10) -1.13* (0.23) -1.79* (0.58)

Outdegree – activity (sqrt) ^1H^ 0.15* (0.03) 0.16* (0.02) 0.30* (0.06) -0.02 (0.08)

^2^ Sex received ^2A^ -0.19* (0.04) -0.05 (0.05) 0.10 (0.06) -0.17 (0.09)

Sex sent ^2B^ -0.02 (0.05) 0.07 (0.05) -0.15 (0.09) -0.72* (0.20)

Sex similarity ^2C^ 0.67* (0.04) 0.84* (0.04) 0.65* (0.07) 0.59* (0.09)

Location similarity ^2C^ 0.43* (0.05) 0.34* (0.05) - -

Class similarity ^2C^ 0.70* (0.05) 0.84* (0.05) 0.94* (0.07) 0.54* (0.11)

Externalizing behavior received ^2B^ 0.03 (0.04) 0.21* (0.04) 0.07 (0.09) -0.03 (0.10)

Externalizing behavior sent ^2A^ 0.12* (0.05) 0.29* (0.04) 0.18 (0.10) 0.72* (0.24)

Externalizing behavior similarity ^2C^ 0.40* (0.18) 1.00* (0.15) 0.60 (0.37) -0.09 (0.43)

Pubertal development received ^2B^ -0.03 (0.02) 0.02 (0.02) -0.08* (0.03) 0.04 (0.04)

Pubertal development sent ^2A^ -0.03 (0.02) 0.01 (0.02) -0.06 (0.04) -0.15* (0.06)

Pubertal development similarity ^2C^ 0.24* (0.11) 0.61* (0.14) 0.55* (0.22) -0.12 (0.30)

Pubertal development sent x 1.03* (0.40) 0.54 (0.31) 1.83* (0.66) 0.19 (0.80)

externalizing behavior similarity maintain ^2D^

Pubertal development sent x -0.42 (0.34) -0.49 (0.30) -0.80 (0.55) 0.19 (0.72)

externalizing behavior similarity create ^2E^

*Behavior Dynamics*

^3^ Externalizing behavior change period 1 ^3A^ 1.33* (0.18) 1.40* (0.26) 1.31* (0.30) 1.48* (0.33)

Externalizing behavior change period 2 ^3A^ 1.54* (0.21) 1.74* (0.32) 1.54* (0.37) 0.99* (0.23)

Externalizing behavior change -1.28* (0.11) -1.19* (0.11) -1.47* (0.22) -1.30* (0.27)

linear shape ^3A^

Externalizing behavior change 0.28* (0.08) 0.13 (0.10) 0.33* (0.15) 0.17 (0.21)

quadratic shape ^3A^

Externalizing behavior influence ^3B^ 1.19* (0.31) 1.06* (0.30) -0.03 (0.85) 1.86 (1.33)

Effect from pubertal development ^3C^ 0.11 (0.09) -0.09 (0.14) 0.32 (0.17) 0.21 (0.23)

Pubertal development x externalizing 0.04 (0.36) 0.85 (0.57) 1.28 (1.32) -2.51 (1.63)

behavior influence ^3D^

*Note. p* < .10 * *p* < .05. ^1^ effects estimating the structure of the friendship network, for descriptions of single effects see the main text. ^2^ effects estimating friendship selection. ^2A^ received effects estimate the number of received friendship ties for participants with this characteristic. ^2B^ sent effects estimate the number of sent out friendship ties for participants with this characteristic. ^2C^ similarity effects estimate if participants base friendship selection on similarity of this characteristic. ^2D^ interaction assessing the impact of pubertal development on likelihood of maintaining friendships based on externalizing behavior. ^2E^ interaction assessing the impact of pubertal development on likelihood of creating friendships based on externalizing behavior ^3^ effects estimating the change of behavior. ^3A^ estimating the development of externalizing behavior, and if this has a linear or quadratic shape. ^3B^ estimating the effect of this characteristic on the development of externalizing behavior. ^3C^ estimating the effect of the average externalizing behavior of friends on the development of participants’ externalizing behavior. ^3D^ interaction assessing the impact of pubertal development on friends’ influence on the development of participants’ externalizing behavior.
